# Spatial–temporal dynamics and driving factor analysis of urban ecological land in Zhuhai city, China

**DOI:** 10.1038/s41598-020-73167-0

**Published:** 2020-09-30

**Authors:** Yunfeng Hu, Yunzhi Zhang

**Affiliations:** 1 State Key Laboratory of Resources and Environmental Information System, Institute of Geographic Sciences and Natural Resources Research, CAS, Beijing, 100101 China; 2 College of Resources and Environment, University of Chinese Academy of Sciences, Beijing, 100049 China

**Keywords:** Ecology, Planetary science

## Abstract

Ecological land is a type of land that has considerable ecological value. Understanding the evolution of urban ecological land in Zhuhai, China, holds great significance for revealing the evolution of ecological land in the Dawan District of southern China. We explored the temporal and spatial variation in urban ecological land in Zhuhai using the transformation matrix, equivalent ecological land, landscape index and ecological land center of gravity migration methods. Multivariate logistic regression was used to analyze the mechanism of ecological land change, and a transition probability map of the ecological land in the study area was drawn. The results showed the following. (1) From 1991 to 2018, the area of ecological land in Zhuhai city continuously decreased, with a reduction in area of 274.8 km^2^, or 32.3%. Sharp changes mainly occurred from 1991 to 2000. (2) The ecological land in the study area has gradually become fragmented, and the degree of landscape heterogeneity has increased. Affected by the expansion of the outer edge of the city to the southwest and the construction of ecological land within the city, the center of gravity of the ecological land has shifted to the northeast by 1346 m. (3) The elevation, slope, distance from built-up land and growth rate of built-up land are important factors influencing the transformation of ecological land. In the future, rivers and shallow coastal waters, tidal flats, and grasslands in the study area have the highest probability of transformation. The Jinwan District and Xiangzhou District will face severe ecological land protection pressure. The method of spatial–temporal analysis of urban ecological land developed in this paper can be applied in similar studies on other cities, and the results obtained for Zhuhai, China, have reference value for future urban planning and ecological protection work.

## Introduction

Land is one of the most important resources on Earth^1^. Over the past 30 years, China has experienced some of the most dramatic and significant changes in land use worldwide. Dramatic land changes have been most prominent in cities and the areas surrounding cities^2,3^ in eastern China (e.g., in the Yangtze River Delta and Bohai Bay regions) and southern China (e.g., in the Dawan District)^4^. Urbanization has completely changed the regional land types, types of ecological services, service quality and service level, resulting in habitat degradation, fragmentation, loss of biodiversity and reduced ecosystem service functions^5^. On a long-term scale, the quantitative and high-precision exploration of the spatial–temporal changes in urban ecological land can clarify the driving mechanism of urban ecological land change, which is essential for sustainable urban development.

Ecological land refers to land resources that provide natural ecosystem services and maintain regional ecological security^6^; it is a land type with ecological functions, and ecological land change is a component of research on land use/cover change^7^. Urban ecology is defined as the organic combination of different types of landscapes, and ecological land is the basis of urban ecological research^8^. The coordinated optimization of the quantity, structure and spatial distribution of land use is key to the rational use of ecological land and the achievement of landscape ecological security^9^. Scholars have studied urban land at different scales and from different perspectives, such as the distribution of urban land use^10^, urban vegetation coverage^11^, urban blue-green space^12^, and urban impervious surfaces^13,14^. Regarding the impacts of urban land use/cover change on the surrounding environment, scholars have studied the impacts of urban vegetation and water bodies on urban heat islands^15,16^, the impacts of urban blue-green space on the health and welfare of urban residents^17^, and the impacts of urban land change on biodiversity^18^. Urban ecological land has important functions in maintaining the stability of ecosystems and providing ecosystem services, and such functions play a vital role in coordinating development and the environment.

The rapid development of remote sensing (RS) Earth observation technology provides abundant, freely available, long-term, medium- and high-resolution satellite imagery resources for urban ecological research. These images can be used to draw long time series and high-precision urban land spatial distribution maps^19,20^. Based on spatial distribution maps of urban land and various spatial analysis models, the spatial patterns and driving forces of urban land changes can be explored, and future development patterns can be predicted^21^. Urban ecological land changes are affected by multiple factors, such as the regional environment, socioeconomics and human factors^22^. Aspects of the regional environment, such as the slope, elevation, soil suitability, accessibility by road and location of urban centers, and socioeconomic factors, such as population, industry, and gross domestic product (GDP), are considered the main drivers^23^. Model analysis methods, such as correlation analysis, stepwise regression analysis, principal component analysis, random forest analysis, and logistic regression analysis, are widely used to explain the driving mechanisms of land use/cover change and to identify the key drivers of such change^24–26^. By monitoring ecological land changes and understanding their impact on the quality of ecological services, a comprehensive evaluation of land changes can be achieved from the dual perspective of quantity and quality. Costanza et al.^27^ proposed the evaluation method and value coefficient of global ecological service value and were the first to realize a quantitative calculation of the value of ecological services. Xie et al.^28^ improved their method based on the characteristics of China's ecological environment and proposed a more practical ecosystem service value per unit area of land. This method is applicable to different ecosystems in China and has been widely used by Chinese scholars^29,30^. The land unit is a mappable landscape unit, and combining changes in the landscape pattern with the quality of ecological services makes it possible to effectively evaluate the impact of land changes on ecological processes and functions^31^. By calculating and analyzing a series of landscape indexes, we can quantitatively evaluate the landscape structure, pattern of ecological land and ecological security of an area. To evaluate ecological processes at the landscape level and to detect and quantify temporal and spatial changes in landscape composition and configuration, researchers have developed a series of indicators, such as patch area, shape, aggregation and diversity, for quantitative analysis^32,33^.

Although the above indicators, models, and methods have been widely used by researchers, to analyze the causes, models, processes and consequences of ecological land change, each method alone is not sufficient, and the applicability of these methods must also be further determined. A single indicator or a small number of indicators cannot provide a complete understanding of a city in terms of quantity, composition, and spatial links^34,35^. Therefore, researchers must integrate existing indicators and methods to gain an overall understanding of urban ecological land change and the future direction of a city.

Since the 1980s, Zhuhai, a typical area in China, has experienced rapid urbanization and dramatic land use changes^36^. Under the influence of the dual pressures of population increase and economic growth, the dynamic changes in and driving factors of suitable ecological land needed to balance Zhuhai's economic development and ecological protection must be analyzed. Exploring the evolutionary process of urban ecological land in Zhuhai holds great significance for revealing the evolutionary law of ecological land in typical urbanization areas in southern China (e.g., the Dawan District). Research in this direction can help determine the ecological value of land, optimize urban ecological land planning, and provide valuable scientific evidence for future urban land planning and policy formulation. To that end, this study selects Zhuhai, China, as a research area. Based on satellite RS image resources from 1991 to 2018, we explore the spatial pattern and evolution of urban ecological land in Zhuhai using RS mapping, geographic information system (GIS) spatial analysis, landscape index analysis, center of gravity migration analysis, and logistic regression analysis. This study attempts to answer the following questions:

1. How has the ecological land in Zhuhai changed since 1991?
2. What are the main factors affecting the change in ecological land in Zhuhai city? What role do these factors play?
3. Where should city policymakers focus in the future, and what kind of ecological land conservation should be performed?

**Figure 1:**
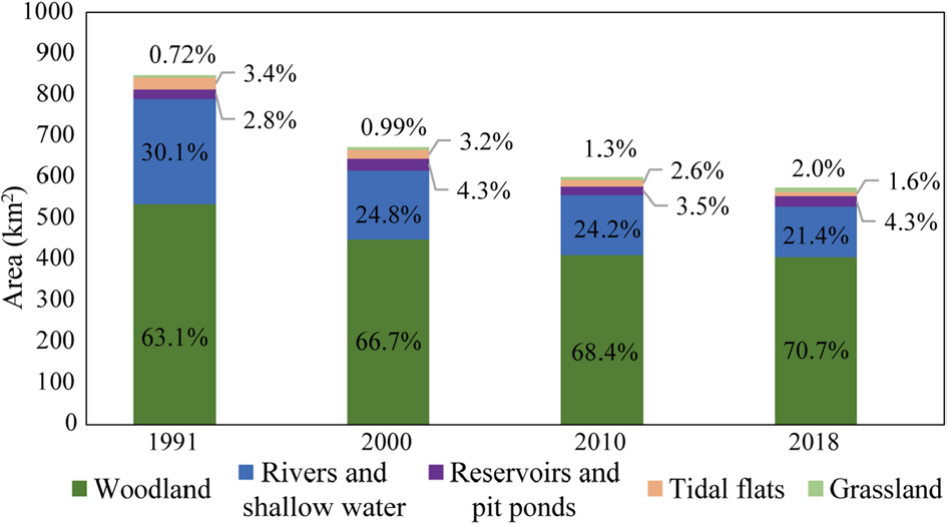
The net change in ecological land in Zhuhai city, 1991–2018. The area of woodland is the largest, followed by the area of rivers and shallow water. The proportions of woodland and grassland in the total area of ecological land increased by 7.6% and 1.3%, respectively. Rivers and shallow water and tidal flats showed downward trends, decreasing by 8.7% and 1.8%, respectively. Reservoirs and pit ponds increased slightly and showed dynamic changes.

**Figure 2:**
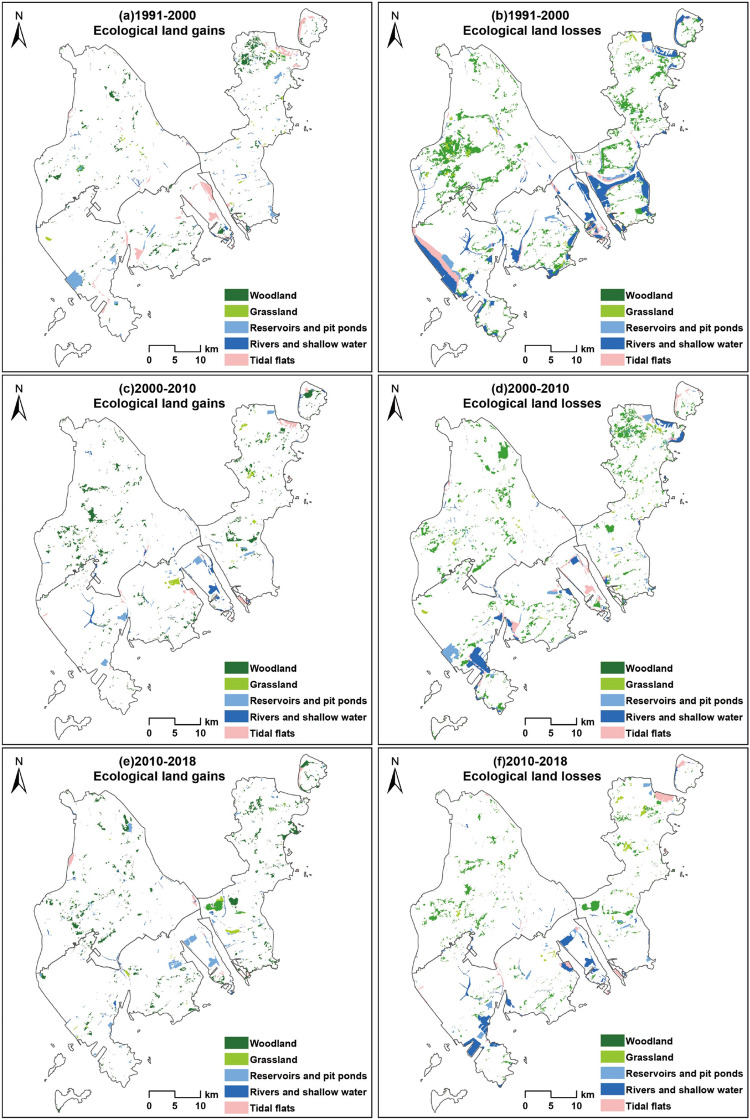
Ecological land gains and losses in Zhuhai city, 1991–2018. (**a**,**c**,**e**) show an increase in ecological land; (**b**,**d**,**f**) show a decrease in ecological land. The decrease in ecological land is obviously higher than the increase, and there is an increase in the degree of patch fragmentation. The reduced patches are mostly marginal woodland and river and shallow water areas. The boundaries of the map come from the Zhuhai Natural Resources Bureau. The drawing of the map was completed with the support of ArcGIS 10.7 software.

**Figure 3:**
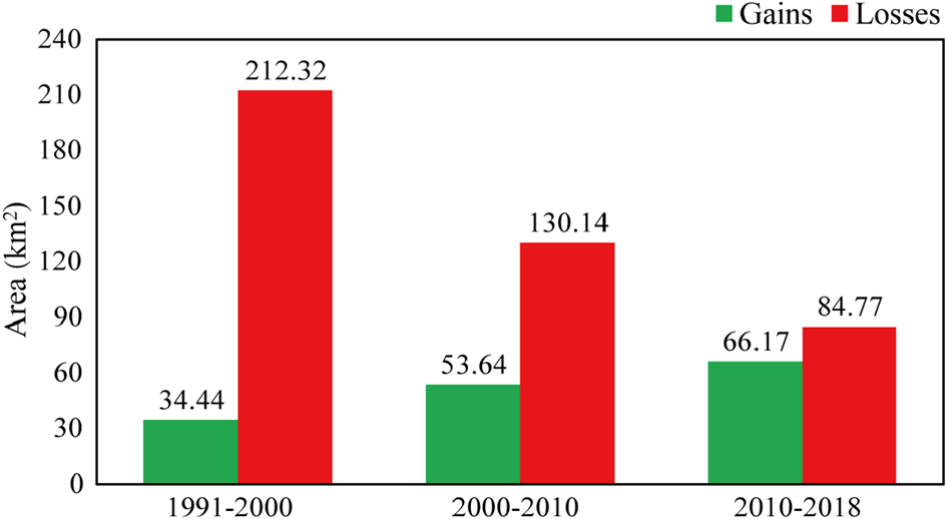
Losses and gains in ecological land area in Zhuhai city, 1991–2018. Green indicates an increase in ecological land, and red indicates a decrease in ecological land. From 1991 to 2000, the net reduction in ecological land was 177.9 km^2^. From 2000 to 2010, the net reduction in ecological land was 76.5 km^2^. From 2010 to 2018, the net reduction in ecological land was 18.6 km^2^.

**Figure 4:**
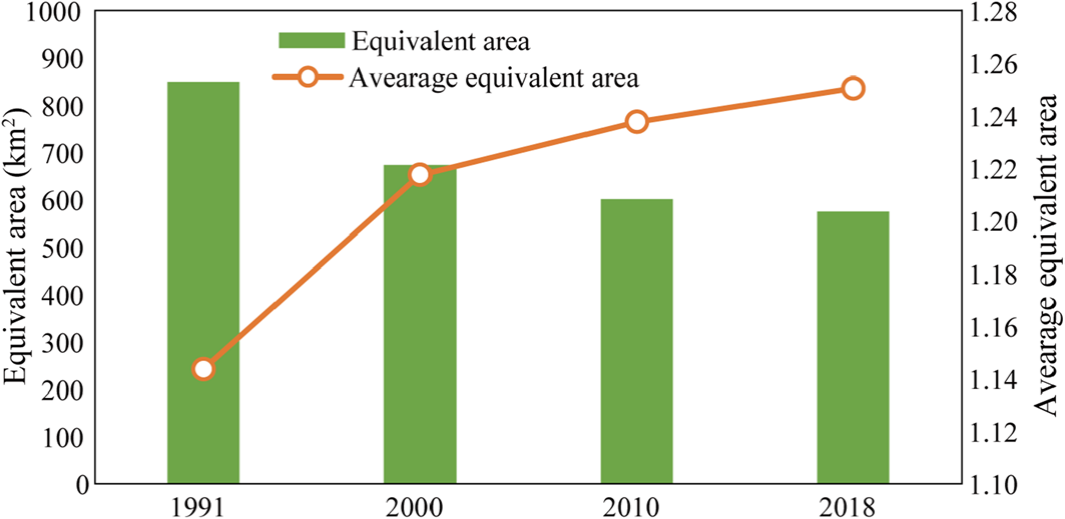
Dynamic changes in ecological land quality in Zhuhai city, 1991–2018. From 1991 to 2018, the equivalent area of ecological land in Zhuhai city showed a downward trend, with a decrease of 274.8 km^2^, i.e., 32.3%. The average equivalent area index showed an upward trend, with an increase of 0.11, i.e., 9.3%.

**Figure 5:**
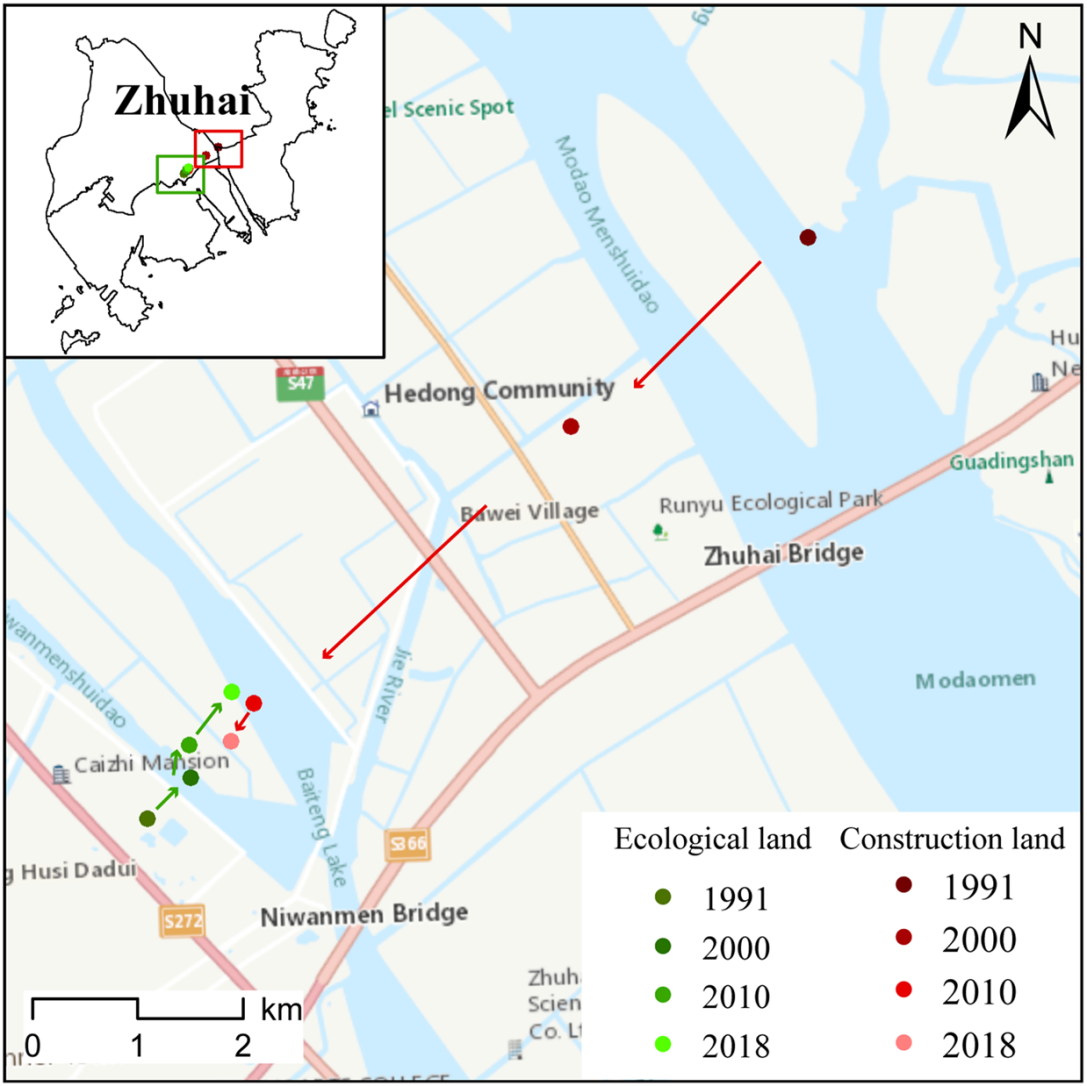
Changes in the center of gravity of ecological land and built-up land in Zhuhai city, 1991–2018. From 1991 to 2018, the center of gravity of ecological land in Zhuhai moved to the northeast by 1346 m. The center of gravity of built-up land moved in the opposite direction, moving 7254 m to the southwest. The boundaries of the map come from the Zhuhai Natural Resources Bureau, and the base map in the main map is the China Online Community Basemap in ArcGIS. The drawing of this map was completed with the support of ArcGIS 10.7 software.

**Figure 6:**
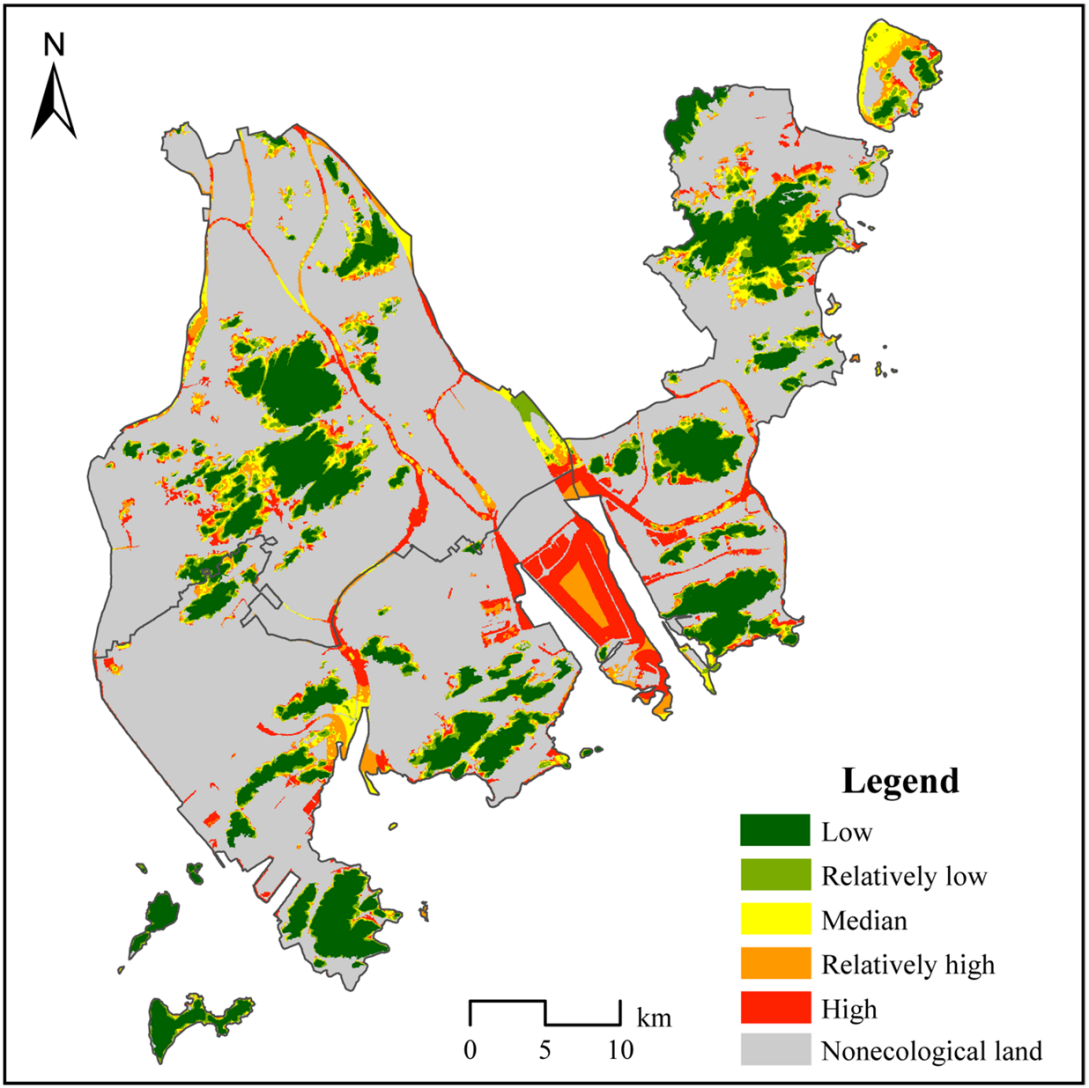
The distribution of the transformation probability of ecological land in Zhuhai city in the future. In Zhuhai, the average transformation probability of ecological land is 0.176; that of forestland is the lowest, at 0.097; that of grassland is 0.257; that of tidal flats is 0.342; that of reservoirs and pit ponds is 0.354; and that of rivers and shallow water is 0.380. The administrative boundary in Fig. 6 comes from the Zhuhai Municipal Bureau of Natural Resources. The goal of the map is to illustrate the probability of the ecological land transformation process. The natural breakpoints method was used to divide the transformation probability of ecological land into 5 levels: a transformation probability less than or equal to 0.1 is defined as low, a transformation probability greater than 0.1 and less than or equal to 0.3 is defined as relatively low, a transformation probability greater than 0.3 and less than or equal to 0.45 is defined as medium, a transition probability greater than 0.45 and less than or equal to 0.65 is defined as relatively high, and a transition probability greater than 0.65 is defined as high. The gray areas in the figure indicate nonecological land.

**Figure 7:**
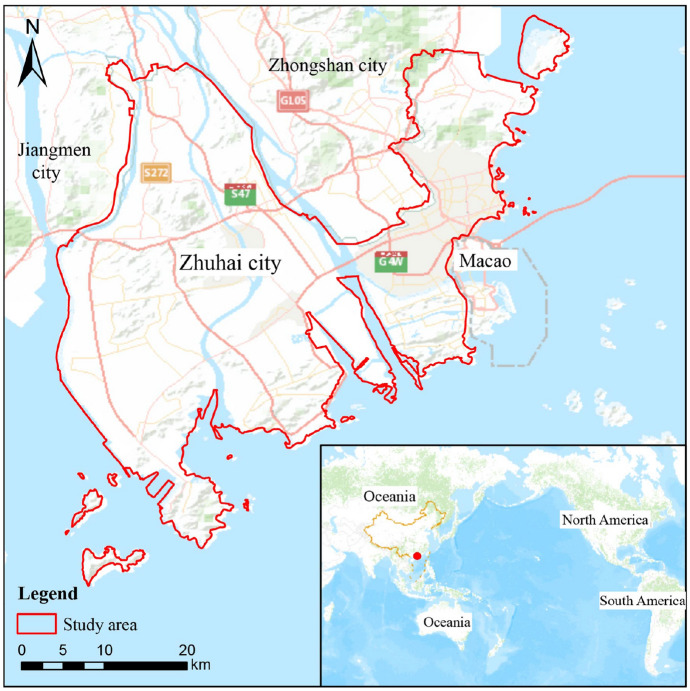
Overview map of the study area. Zhuhai city is the core city of the Dawan District and the Pearl River Estuary in southern China. It is connected to Macao to the south and Hong Kong to the east. It covers an area of 1732 km^2^, and it has a total population of 1.57 million, a sea area of 5941.8 km^2^, a coastline that is 224.5 km long, and 217 large and small islands. Additionally, it has jurisdiction over three administrative districts: Xiangzhou, Doumen and Jinwan. The boundaries of the map come from the Zhuhai Natural Resources Bureau, and the base map is the China Online Community Basemap in ArcGIS. The drawing of the map was completed with the support of ArcGIS 10.7 software.

It is worth mentioning that all maps (Figs. 2, 5, 6 and 7) were drafted by the authors using ArcGIS V10.7 (https://www.esri.com) on a personal computer, and all charts (Figs. 1, 3 and 4) were created using MS Excel 2013 (https://www.microsoft.com).

## Results

### Land use mapping and accuracy assessment

According to the land use planning map of Zhuhai city, the characteristics of the city, the status of human activities and land use, and the types of natural ecosystems, we identified and categorized land use into 10 types: woodland, grassland, rainfed cropland, paddy fields, aquaculture areas, reservoirs and pit ponds, tidal flats, rivers and shallow water, built-up land and unutilized land (Supplemental Materials S1: Land use types and descriptions). The ecological land types include woodland, grassland, reservoirs and pit ponds, tidal flats, and rivers and shallow water. Rainfed cropland, paddy fields and aquaculture areas were not included as ecological land types because they are agricultural land mainly used for agricultural production. These land use types are greatly disturbed by humans, their ecological functions are very fragile, and they are affected by economic interests and have low ecological value. Unutilized land provides few ecological benefits and may be converted into built-up land in the short term; thus, its ecological benefits are unsustainable.

After the preprocessing and splicing of multiperiod satellite RS images, we completed object-based multiscale automatic segmentation and land use classification of the images using eCognition Developer software. Specifically, the Estimation of Scale Parameters (ESP) tool was first used to obtain the local variance parameter, which reflects the internal homogeneity of the segmentation object; then, the rate of change (ROC) of the local variance (LV) parameter was calculated^37,38^. When the ROC reaches its peak, the corresponding segmentation scale can be used as the optimal segmentation scale^37^. At the optimal segmentation scale, classification is based on the object unit using the nearest neighbor method of eCognition Developer. The nearest neighbor method is a commonly used supervised classification method that is simple and easy to understand, and it is suitable for multiclassification problems^39^.

Finally, based on the preliminary results data of the four stages automatically classified by eCognition Developer, obvious errors and omissions in the data of the preliminary results were revised and improved through manual visual interpretation. The final revised result data were used for the subsequent analysis of the land pattern and its changes.

This study first drew land use maps for four years: 1991, 2000, 2010, and 2018. We extracted no less than 200 regions of interest (ROIs) in each study year and compared high-resolution Google Earth images to perform a land mapping accuracy assessment. To ensure that the accuracy of each land type was reliably estimated, we confirmed that each land type had at least 10 ROIs when laying out the ROI area. Table 1 shows the land use classification accuracy for the 1991–2018 period. The overall accuracy of the land mapping for 1991, 2000, 2010, and 2018 was 93.4%, 94.1%, 91.1%, and 94.5%, respectively, and the Kappa coefficients were 0.925, 0.933, 0.890, and 0.938, respectively, meeting the research requirements.

**Table 1:** Classification accuracy of land use types in Zhuhai city.

Year	1991	2000	2010	2018
Overall accuracy (%)	93.40	94.07	91.10	94.53
Kappa coefficient (KA)	0.9250	0.9330	0.8995	0.9378

### Spatial patterns and dynamics of ecological land

From 1991 to 2018, the ecological land in Zhuhai was dominated by woodland and rivers and shallow water, and the overall area of ecological land continuously decreased (Fig. 1). In 1991, the total area of ecological land was 849.4 km^2^, accounting for 53.7% of Zhuhai's urban area. In 2018, the area was reduced to 574.6 km^2^, accounting for only 36.3% of Zhuhai's urban area.

In 28 years, the amount of ecological land decreased by 32.3%, of which woodland decreased by 24.2% (129.6 km^2^), tidal flats decreased by 67.2% (19.3 km^2^), and rivers and shallow water decreased by as much as 51.8% (132.3 km^2^). The reduction in rivers and shallow water represented the bulk of the reduction in ecological land area (48.1%). In contrast, the area of reservoirs and pit ponds grew slightly while maintaining a steady state, increasing by 1.1 km^2^. Compared with 1991, the grassland area grew slightly, increasing by 5.3 km^2^, mainly due to the construction of golf courses and parks. Clearly, there is an order of magnitude difference between the increase and decrease in ecological land.

From the temporal perspective (Fig. 2), the change in ecological land mainly occurred in the 1991–2000 period. During this period, the reduction in ecological land was the largest (212.3 km^2^), mainly distributed in the contiguous area of woodland and built-up land in the central and western areas of the Doumen District and in the coastal areas of the Jinwan District and Xiangzhou District. At the same time, there was a small increase in ecological land, mainly due to the restoration and regulation of tidal flats and reservoirs and pit ponds.

Since 2000, ecological environmental protection and construction work have gradually been taken more seriously, and the State Council of China promulgated the "National Ecological Environmental Protection Program". Local governments at all levels have gradually strengthened their awareness of ecological environmental protection. The occupation of ecological land by urban development has rapidly decreased, while the area of new ecological land formed by ecological protection and ecological restoration has gradually and steadily increased. From 2000 to 2010, the ecological land in Zhuhai decreased by 130.1 km^2^ and increased by 53.6 km^2^, with a net reduction of 76.5 km^2^. From 2010 to 2018, the decrease and increase in ecological land were similar, and the net reduction in area was only 18.6 km^2^; thus, the spatial distribution and quantity of ecological land in Zhuhai city was approximately stable (Fig. 3).

In the 28-year monitoring period of this paper, the reduction in ecological land in the first 10 years (1991–2000) was 0.99 times that in the subsequent 18 years (2000–2018). The total amount of ecological land added in the subsequent 18 years (2000–2018) was 3.5 times that of the first 10 years (1991–2000).

### Landscape characteristics

At the landscape level (Table 2), the edge density (ED) of ecological land in the study area is significantly lower than that of nonecological land. The ED exhibited a pattern of first increasing, then decreasing, and subsequently slightly increasing (with values of 33.6 in 1991, 37.7 in 2000, 31.8 in 2010, and 34.7 in 2018). The patch density (PD), landscape shape index (LSI), and largest patch index (LPI) had the same trend as that of the ED. These changes indicate that over time, the landscape of ecological land began to experience an increase in fragmentation and a decrease in regularity and continuity; then, the landscape was reintegrated into a more regular and continuous pattern.

**Table 2:** Changes in landscape-level indexes in Zhuhai city, 1991–2018.

Class	Year	ED (m/hm^2^)	PD (pcs/100 hm^2^)	LSI (%)	LPI (%)	CONTAG (%)	SHDI	SHEI
Ecological land	1991	7.0	0.58	29.2	17.8	70.0	0.90	0.56
	2000	7.1	1.0	34.9	10.7	69.5	0.92	0.57
	2010	4.9	0.74	32.5	9.9	71.3	0.88	0.54
	2018	5.2	0.89	32.8	10.8	71.7	0.86	0.53
Nonecological land	1991	33.7	1.2	44.4	19.3	50.8	1.34	0.83
	2000	31.1	1.1	46.7	10.0	55.0	1.22	0.76
	2010	23.2	0.63	39.3	11.2	52.1	1.35	0.84
	2018	27.5	0.68	42.6	7.0	53.1	1.29	0.80
All land	1991	33.6	0.86	37.5	9.6	55.2	1.79	0.78
	2000	37.7	1.1	41.5	5.8	55.1	1.77	0.77
	2010	31.8	0.67	35.6	7.0	54.4	1.83	0.80
	2018	34.7	0.76	38.6	4.5	54.9	1.79	0.78

At the landscape level, ED is the edge density, PD is the patch density, LSI is the landscape shape index, LPI is the largest patch index, CONTAG is the contagion index, SHDI is Shannon’s diversity index, and SHEI is Shannon’s evenness index.

In addition, from 1991 to 2018, the contagion index (CONTAG) of all land in Zhuhai city fluctuated slightly at approximately 55%, and the degree of landscape pattern aggregation did not change much. However, the CONTAG of ecological land was approximately 70%, which was significantly higher than that of nonecological land; this result indicates that the CONTAG and connectivity of ecological land were higher than those of nonecological land. Shannon’s diversity index (SHDI) and Shannon’s evenness index (SHEI) did not change much in the time series, indicating that the landscape diversity of Zhuhai city has basically been stable over the past 28 years. However, compared with 1991, the SHDI and SHEI decreased slightly, indicating that the ecological landscape diversity and uniformity decreased in the study area, while the landscape heterogeneity increased.

At the class level (Table 3), the PD and the area-weighted mean contiguity index (CONTIG_AM) of woodland remained basically unchanged, the LSI increased from 19.99 to 21.7, and the LPI decreased from 9.6 to 3.9. These changes were caused by the following processes: the expansion of built-up land, the preferential occupation of marginal forestland by built-up land, the reduction in the dominance of the landscape type, and the increasing complexity of the original geometry. However, woodland mainly exists in a continuous form, and these encroachment behaviors have little effect on the number, spatial connectivity or proximity of woodland patches.

**Table 3:** Changes in class-level indexes in Zhuhai city, 1991–2018.

Land use type	PD (pcs/100 hm^2^)		LSI (%)		LPI (%)		CONTIG_AM	
	1991 year	2018 year	1991 year	2018 year	1991 year	2018 year	1991 year	2018 year
Woodland	0.08	0.08	20.0	21.7	9.6	3.9	0.97	0.96
Grassland	0.02	0.01	11.4	7.2	0.04	0.31	0.84	0.93
Reservoirs and pit ponds	0.07	0.07	14.1	16.3	0.20	0.15	0.90	0.89
Tidal flats	0.04	0.01	14.3	8.0	0.82	0.12	0.91	0.91
Rivers and shallow water	0.11	0.15	22.8	26.4	6.9	1.4	0.95	0.92

At the class level, PD is the patch density, LSI is the landscape shape index, LPI is the largest patch index, and CONTIG_AM is the area-weighted mean contiguity index.

The PD and LSI of grassland showed downward trends, while the LPI and CONTIG_AM showed upward trends. This result is closely related to the increase in grassland in the study area. The increased grassland caused the number of patches to increase slightly, improving the superiority of the landscape. The construction of artificial grassland is more regular in the shape of grass patches, and the connectivity is enhanced between landscape units.

In addition, the PD, LSI and LPI of tidal flats showed downward trends, indicating that the development and utilization of tidal flat reclamation were strengthened, the number decreased, and the shape tended to be regular. The landscape characteristics of reservoirs and pit ponds and rivers and shallow water were basically the same: the LSI showed an upward trend, indicating that the patches were seriously disturbed by human activities, the large patches experienced continuous fragmentation, and the landscape type shapes were complicated. In contrast, the LPI showed a downward trend, indicating that activities such as sea filling led to a continuous decrease in sea area.

### Ecological quality evaluation

Ecological quality is used to characterize the conditions of the ecosystem; the ecosystem is disturbed by human activities and land use change, and the ability to provide services is also affected^40^. The value of ecosystem services is an important comprehensive indicator reflecting ecological quality, and the ecological service value of ecological land is higher than that of nonecological land^41^. Based on the ecosystem service value coefficient proposed by Xie et al.^28^, we normalized the coefficient value to 0–1 and used the equivalent area and the average equivalent area, which were used to evaluate the ecological service quality of ecological land.

The transformation matrix of ecological land and nonecological land shows the following (Table 4): the probability of ecological land being transformed into nonecological land in the periods 1991–2000, 2000–2010 and 2010–2018 was 25.0%, 19.4% and 14.3%, respectively. The contributions of ecological land to nonecological land were 23.3%, 13.2% and 8.4%, respectively. The transformation of ecological land to nonecological land showed a weakening trend after 2000, and the ecological quality showed improvement.

**Table 4:** Probability of ecological land being transformed into nonecological land in Zhuhai city, 1991–2018.

Type	1991–2000		2000–2010		2010–2018	
	Ecological land	Nonecological land	Ecological land	Nonecological land	Ecological land	Nonecological land
**Ecological land**						
B (%)	94.9	23.3	91.0	13.2	88.5	8.4
C (%)	75.0	25.0	80.6	19.4	85.7	14.3
**Nonecological land**						
B (%)	5.1	76.7	9.0	86.8	11.5	91.6
C (%)	4.7	95.3	5.9	94.1	6.7	93.3

B is the contribution of a land type to another land type, and C is the probability of a transition from one land type to another.

From 1991 to 2018, the equivalent area of ecological land continued to decrease, but the downward trend gradually stabilized after 2000 (Fig. 4). In 1991, the equivalent area of regional ecological land was 849.4 km^2^, and in 2000, it was 673.2 km^2^, indicating a significant decrease in the equivalent area, with a reduction of 20.7%. In 2010, the equivalent area of ecological land further dropped to 600.2 km^2^, a reduction of 10.8%, although the decrease was significantly smaller than that in the previous period. In 2018, the equivalent area was 574.6 km^2^, representing a reduction of only 4.3%.

As shown in Fig. 4, the average equivalent area of ecological land showed a continuous upward trend. Specifically, the average equivalent area was 1.14 in 1991, 1.22 in 2000, 1.24 in 2010, and 1.25 in 2018. This result shows that although the ecological land area decreased, the quality of the ecological land gradually improved. In reality, this pattern was manifested as follows: the area of grasslands and reservoirs and pit ponds gradually increased, the degree of landscape fragmentation weakened, and the landscape dominance became more obvious. In addition, these land types have relatively high ecosystem service values among all land types.

### Changes in the center of gravity of ecological land

From 1991 to 2018, the center of gravity of ecological land shifted to the northeast, and the center of gravity of built-up land shifted to the southwest (Fig. 5).

From 1991 to 2000, the center of gravity of ecological land moved 404 m to the east and 409 m to the north, and the overall movement was 578 m to the northeast. From 2000 to 2010, the center of gravity of ecological land moved 24 m to the east and 355 m to the north, and the overall movement trend was northward. From 2010 to 2018, the center of gravity of ecological land moved 273 m to the east and 236 m to the north, and the overall movement was 473 m to the northeast. In these three periods, the center of gravity of built-up land moved to the southwest by 2871 m, 3983 m and 424 m. The urban expansion and internal construction mainly experienced a rapid and then slow evolution from the northeast to the southwest.

From the spatial distribution of all ecological land types, the center of gravity of woodland moved to the southeast (0.68 km) from 1991 to 2018. This movement occurred because human construction activities such as deforestation, urban expansion, and infrastructure construction were prominent in the western and northern parts of Zhuhai during the 1991–2000 period. The movement of the center of gravity of grassland to the east and south was highly related to the construction of golf courses, such as the Zhuxiandong Golf Club in the Xiangzhou District, the Dananshan Cuihu Golf Course in Jinding Town, a golf club in the Jinwan District, and Zhuhai Stadium in the Xiangzhou District. The center of gravity of reservoirs and pit ponds moved southward (2.9 km); the center of gravity of tidal flats moved eastward (5.8 km); and the center of gravity of rivers and shallow water moved northward (3.5 km). These changes were closely related to the reclamation engineering carried out by Zhuhai city in recent years.

### Modeling the ecological land change process

Changes in urban ecological land are mainly due to the expansion of the outer edge of cities and the oppression of urban internal land development. Therefore, we selected four indicators of natural geography and regional development that might reflect changes in urban expansion and urban construction: elevation, slope, distance from built-up land, and growth rate of built-up land.

With the support of SPSS software, the equation of the transformation probability of ecological land to nonecological land in Zhuhai can be obtained through the binary logistic regression analysis module. Specifically, this equation is expressed as follows (see Supplemental Materials S2: Parameter of the driving factors for modeling):

1 $$P = 1 - \frac{1}{{{1 + }e^{{{ - }\left( {{0}{\text{.069}} \times {\text{A } + \text{ 0}}{.033} \times {\text{B } + \text{ 0}}{.473} \times {\text{C } - \text{ 1}}{.079} \times {\text{D } - \text{ 0}}{.963} \times {\text{E } - \text{ 0}}{.853}} \right)}} }}$$

where *A* is the slope; *B* is the elevation; *C* is the distance from built-up land; and D and E are the built-up land growth rates of categories 4 and 5, respectively. The squared maximum likelihood of the numerical values (− 2 log-likelihood) of the model was 18,155.4, and the value of the χ^2^(5) comprehensive test statistic was 7871.2 (p < 0.001), which was significantly higher than the critical test value of 20.5. This result shows that the model constructed based on the above training data has superior precision and can be used for future prediction simulations.

To eliminate the influence of the distribution deviation of the training samples, we performed model accuracy verification. We randomly selected 10,000 ecological land sample points from the land use map for 1991 and observed their changes in 2018; we then tested the simulation accuracy of the model. The results show (Table 5) that there are 7853 sample points with the same simulation results as the actual observations. Thus, the model simulation accuracy is 78.6%, indicating that the model has high accuracy and robustness.

**Table 5:** Logistic model estimation accuracy: a total of 10,000 sample points with an overall accuracy of 78.6%.

Observation value	Predictive value		
	Unchanged	Changed	Accuracy
**Ecological land**			
Unchanged	5230	1184	81.5%
Changed	963	2623	73.1%
Overall accuracy			78.6%

### Transformation probability of ecological land

Based on the above model, elevation, slope, distance from built-up land (based on 2018 data) and growth rate of built-up land (based on 2010–2018 change data) are used as input variables to predict the future transformation probability of ecological land to nonecological land in Zhuhai city (Fig. 6).

The simulation results show that the average transformation probability of ecological land in Zhuhai is 0.176. The transformation probabilities of tidal flats, reservoirs and pit ponds, rivers and shallow water and grassland (0.342, 0.354, 0.380, and 0.257, respectively) are higher than the average transformation probability of ecological land in the study area, while the transformation probability of woodland is 0.097, which is significantly lower than the average transformation probability of regional ecological land. The reasons for these patterns are as follows: tidal flats, reservoirs and pit ponds and grassland are distributed in flat areas with low elevations, meaning that it is easier to prioritize their development and utilization. In addition, because Zhuhai is a coastal city, urban builders have a strong impulse to reclaim land, which is likely to result in the transformation of ecological land in the form of tidal flats and rivers and shallow water to built-up land. On the other hand, woodland in the study area is mostly in areas with relatively steep slopes and high elevations, mainly including Huangyang Mountain, the Fenghuang Mountain Nature Reserve, Jianfeng Mountain and Lanlang Mountain Forest Park. These woodlands are less likely to be further developed and utilized.

In terms of spatial distribution, ecological land in the Jinwan District has the highest transformation probability, followed by that in the Xiangzhou District. The ecological land in the Doumen District, an important agricultural product protection and ecological agricultural development zone in Zhuhai, is mostly unsuitable for woodland development. Therefore, the ecological land in this district has the lowest transformation probability, and there is less pressure affecting ecological land protection. The Jinwan District and Xiangzhou District are zones in Zhuhai that gather high-end industries and business services, and there is a strong demand for land for regional development. In addition, these two districts are coastal and riverside areas; thus, city planners and builders have a strong impulse to expand urban built-up land by means of sea reclamation and river reconstruction. In these two districts, the pressure on regional ecological land protection is enormous, and the need to apply new technologies and methods to save land and increase ecological land is the strongest.

## Discussion

### Quick and accurate land use mapping is the basis of ecological land change assessment

Many RS interpretation algorithms have been developed to extract land cover and land use types for urban land mapping and include deep learning^42^, neural networks^20^, support vector machines (SVMs)^43^, and decision trees^44^. eCognition Developer software and the Google Earth Engine (GEE) also facilitate automatic land use classification^45,46^, but automatic classification still has certain limitations. In particular, when analyzing dynamic changes, errors in land classification may spread to dynamic quantification^47^. In this study, manual visual means were used to correct the results of the automatic classification, which ensured the accuracy of the mapping and made the overall accuracy higher than 90%. However, the use of such means may lead to high costs in large-scale regional studies. Urban ecological land types can involve a more sophisticated classification; for example, different types of forestland and water, such as shrubs, orchards, rivers and beaches, may have great differences in terms of their ecological value. In future research, we can further explore the full use of data such as high-resolution RS images and urban function hotspot data to improve the fine-grained division of land use types and their ecological functions.

### There are great uncertainties in research on the driving mechanism of land use change

Multivariate logistic regression has been widely used in land use change studies^48^, for example, to extract the driving factors of and to simulate the future scenarios of regional land use change^49^. However, a logistic regression model can explain the main influencing factors only in a specific period, and it is difficult to accurately simulate and predict long-term and dynamic change^50^. The optimization and improvement of models and algorithms must be further studied, and new algorithms, such as generalized cross decomposition^51^, generalized outer approximation^52^ and selective maintenance scheduling with multiple maintenance actions^53^, can be introduced to solve complex nonlinear problems and improve the applicability and accuracy of simulation prediction. In the current study, we considered only four explanatory variables of driving factors, and some important natural and social factors, such as precipitation, meteorological disasters, geological structures, geological disasters, and functional types of urban blocks, may have been omitted. We sincerely hope that more scholars will make breakthroughs and progress in regard to these aspects in the future.

### Managerial implications for the government

For a long time, Chinese local governments and people have not fully understood the role and value of urban ecological land^54^. Local governments pay more attention to economic development than to the maintenance of a good urban ecology, which readily leads to ecological land being occupied in the process of land use planning and management^55^. Our study shows that from 1991 to 2018, the ecological land area of Zhuhai continuously decreased and that landscape fragmentation and heterogeneity increased. The rapid change in land mainly occurred from 1991 to 2000. In the future, tidal flats and rivers and shallow water have the highest probability of transformation in the study area. The Jinwan District and Xiangzhou District face severe pressure in terms of ecological land protection. The results regarding historical changes and future trends pose serious challenges to rational urban land planning and sustainable urban construction for Zhuhai authorities, and they also provide the government with key ecological land types and key spatial places for protection efforts in urban ecological land.

## Conclusions

With the support of RS and GIS technology, we applied the transformation matrix method, equivalent ecological land method, landscape index method, center of gravity migration method, logistic regression modeling and simulation prediction method to conduct multidimensional and comprehensive research on the changes in ecological land in Zhuhai, China. We constructed a complete technical route for urban ecological land assessment. This technical route comprehensively applied Earth observation and cloud storage, cloud computing technology, satellite image automatic classification technology, and GIS spatial analysis technology, as well as the ecological landscape index method, logistic model construction method, and land transformation probability simulation prediction method, and it realized a comprehensive and systematic analysis of ecological land from the historical evolution of the spatial–temporal trajectory to the driving mechanism of change to future prediction. This technical method can be applied in similar research on other regions and cities around the world, and the results of this paper can provide a scientific basis for urban planning and ecological protection for the Zhuhai government and even other large municipalities in southern China.

## Materials and methods

### Study area

Zhuhai city is located at 21° 48′–22° 27′ N and 113° 03′–114°19′ E (Fig. 7). Zhuhai faces Hong Kong across the sea to the east, Macao to the south, Jiangmen city to the west and Zhongshan city to the north. Zhuhai is an important gateway connecting Guangdong, Hong Kong and Macao. It is the core city of the Dawan District in southern China and is located on the western bank of the Pearl River Estuary. The total population of Zhuhai city is 1.57 million, and it has jurisdiction over three administrative districts: Xiangzhou, Doumen and Jinwan. The landforms are diverse, mainly consisting of plains and low hills with terraces and tidal flats. The sea area of Zhuhai city is 5941.8 km^2^, the coastline is 224.5 km long, and there are 217 large and small islands. The city has the largest sea area and the largest number of islands among the cities of the Pearl River Delta. The river network in the territory of Zhuhai is dense and braided. Zhuhai has a subtropical maritime climate, and the vegetation type is tropical evergreen forest. The annual average temperature of the study area is 22.4 °C, and the average annual rainfall is 1700–2300 mm.

In this study, the study area was limited to the land area of Zhuhai city. The scope of the study includes continental coastal tidal flats but does not include the Wanshan Islands, which are scattered in the outer sea.

### Basic supporting datasets

The data involved in this study include Landsat RS image data, digital elevation model (DEM) data, and Zhuhai administrative division and related land planning data. Among them, Landsat Thematic Mapper/Enhanced Thematic Mapper Plus (TM/ETM+) and Landsat Operational Land Imager (OLI) data for 1991, 2000, 2010 and 2018 were obtained from the United States Geological Survey (USGS) website (https://earthexplorer.usgs.gov/). For details on the data, see Supplemental Materials S3. Based on satellite RS images, land use classification and mapping can be conducted.

The DEM is a ground model that represents ground elevation in the form of an ordered array of numerical values. For the study area, we selected ASTER GDEM DEM data; these data have a spatial resolution of 30 m and were derived from the Geospatial Data Cloud Platform (https://www.gscloud.cn/search). Based on the DEM data, derived indicators, such as slope, can be extracted.

The 2006 land use map and overall land use planning map (2006–2020) for Zhuhai came from the Zhuhai Natural Resources Bureau (https://www.gtjzh.gov.cn/gtxx/gtzylygh/). The scale of the map was 1:350,000, and the map was compiled by the Zhuhai Municipal Bureau of Land and Resources and the Land Research Center of Sun Yat-sen University. With the support of GIS software, automatic classification data can be corrected based on urban land use data, and additional data such as the distance from urban built-up land can be calculated.

### Land transformation matrix

The land change transformation matrix is used to quantitatively describe the number of land use type transformations in closed land systems^56^. The calculation formula of the change amount of a certain type of land transformation is as follows:

2 $$A = A_{b} - A_{a}$$

where *A* is the amount of change in a land type, and *A*_*a*_ and *A*_*b*_ are the areas of the land type at the beginning and end of the study period, respectively.

Similar to the land change transformation matrix, the land transformation probability matrix is composed of transition probabilities, i.e., the probability of one land type being converted into another land type. The transition probability matrix can reveal the mutual transformation status and transition probability between different types of land^57^. The formula is as follows:

3 $$P = P_{ij} = \left[ \begin{gathered} P_{11} \;P_{12} \; \cdots \;P_{1n} \hfill \\ P_{21} \;P_{22} \; \cdots \;P_{2n} \hfill \\ \cdots \;\; \cdots \;\; \ddots \;\; \cdots \hfill \\ P_{n1} \;P_{n2} \; \cdots \;P_{nn} \hfill \\ \end{gathered} \right]$$

where n is the number of land types and *P*_*ij*_ is the transition probability of land type i to type j. Each element in the matrix must meet the following two conditions:

4 $$0 \le P_{ij} \le 1,\quad \sum\limits_{j = 1}^{n} {P_{ij} = 1}$$

The state of the land type at any time is determined by the initial state and the transition probability. The state of a land use type at start time t and end time t + 1 can be expressed as follows:

5 $$S_{t + 1} = P_{ij} \times S_{t}$$

where *S*_*t*_ and *S*_*t*+*1*_ are the area or state probabilities of the land type at time t and time t + 1, respectively.

### Landscape ecological index

Landscape ecology refers to the composition, diversity, shape and spatial pattern of landscape structural units. Differences in the types, shapes, sizes, quantities and spatial combinations of landscape patches reflect differences in the quality of landscape functions and the ecological processes of the entire region^58^. In this study, FRAGSTATS 4.2 was used to calculate the landscape index. This software can calculate the landscape pattern index from three levels: the landscape level, class level and patch level^59^. This paper explores the evolution of the landscape spatial patterns in Zhuhai from the class and landscape levels.

There were five indicators at the class level, including the PD, LSI, LPI, and CONTIG_AM. The seven indicators at the landscape level included the ED, PD, LSI, LPI, CONTAG, SHDI, and SHEI. The calculation methods and ecological meanings of each index are based on the common formulas and expressions used by scholars in China and other countries^33^ (see Supplemental Materials S4: Landscape indicators and descriptions).

### Equivalent ecological area

The global ecological service value assessment method and value coefficient proposed by Costanza et al.^27^ realized the first quantitative calculation of the value of ecological services. Based on their method, Xie et al.^28^ further determined the value of ecosystem services per unit area of land for different ecosystems in China.

In this study, we first merged and reclassified the land use types of Zhuhai according to the closest ecosystem type. Then, we considered that the service value (absolute value) of the unit ecosystem area lacked the promotion value globally and in other regions of the country. According to the relative proportional relationship between the service value (absolute value) of different ecosystems, the values were normalized to values between 0 and 1 (i.e., each value became a relative value)^60^ (Table 6). Finally, we multiplied the abovementioned ecological service value (relative value) by the area of the corresponding ecosystem type to obtain the equivalent ecological area. The calculation formula is as follows:

6 $$Q = \frac{{\sum\nolimits_{i = 1}^{n} {\delta_{i} \times A_{i} } }}{A}$$

**Table 6:** Normalized ecosystem service value of each land use type in Zhuhai city.

Land types	Woodland	Grassland	Rainfed cropland	Paddy fields	Aquaculture areas	Reservoirs and pit ponds	Tidal flats	Rivers and shallow water	Built-up land	Unutilized land
Normalized ecosystem service value	1.00	0.47	0.16	0.29	0.23	0.63	0.72	0.92	0.08	0.10

The values refer to the ecosystem service value equivalence factor and the relative proportional relationship between the value of the ecosystem services of different ecosystems, normalized to 0–1, and we performed a re-evaluation of the ecological service value of each land type. The value of woodland is the highest (1.0), while that of unutilized land is the lowest (0.1).

where *Q* is the average equivalent ecological area, *δ*_*i*_ is the normalized value of the ecosystem service value of land type i, *A*_*i*_ is the area of land type i, *A* is the total area of all land types, and *n* is the number of land use types.

### Center of gravity transformation model

The center of gravity transformation model can adequately describe the spatial evolutionary processes of populations, economic activities, and land distribution patterns^61^. In this study, the calculation method of the position of the center of gravity of ecological land is as follows:

7 $$X_{t} = \sum\nolimits_{i = 1}^{n} {\left( {C_{ti} \times X_{i} } \right)}/\sum\limits_{i = 1}^{n} {C_{ti} }$$

8 $$Y_{t} = \sum\nolimits_{i = 1}^{n} {\left( {C_{ti} \times Y_{i} } \right)}/\sum\limits_{i = 1}^{n} {C_{ti} }$$

where *X*_*t*_ and *Y*_*t*_ are the latitude and longitude coordinates, respectively, of the center of gravity of ecological land in year t, *C*_*ti*_ is the area of the i-th regional ecological land, and *X*_*i*_ and *Y*_*i*_ are the latitude and longitude coordinates, respectively, of the geometric center of the i-th region.

### Logistic regression model

The results of land use change can be divided into two states, change or no change; thus, these states can be represented by the numbers 0 or 1, respectively. A logistic regression model can be used to construct the relationship between multiple explanatory variables and one response variable (binary variable)^62^.

Let the independent variable x_i_ = (x_1i_, x_2i_, x_ki_) of the i-th case and the dichotomous variable yi take the value of 0 or 1 (y_i_ = 0 means that the event does not occur; y_i_ = 1 means that the event occurs). The conditional probability of the occurrence of an event can be denoted as follows:

9 $$P_{i} = P\left( {y_{i} = 1|X_{i} } \right) = \frac{{1}}{{{1 + }e^{{ - \left( {\alpha + \sum\nolimits_{j = 1}^{k} {\beta_{j} x_{ji} } } \right)}} }}{ = }\frac{{e^{{\left( {\alpha + \sum\nolimits_{j = 1}^{k} {\beta_{j} x_{ji} } } \right)}} }}{{1 + e^{{\left( {\alpha + \sum\nolimits_{j = 1}^{k} {\beta_{j} x_{ji} } } \right)}} }}$$

Therefore, the binary logistic regression model can be expressed as follows:

10 $$Log\;it\left( {P_{i} } \right) = \ln \left( {\frac{{P_{i} }}{{1 - P_{i} }}} \right) = \alpha + \beta_{1} x_{1i} + \beta_{2} x_{2i} + \cdots + \beta_{k} x_{ki}$$

where *P*_*i*_ is the probability of the event occurring in the i-th case, *α* is a constant term, *β*_*k*_ is a regression coefficient, and the nonlinear function is composed of k explanatory variables.

The conditional probability of the nonoccurrence of an event can be expressed as follows:

11 $$1 - Pi = 1 - \frac{{e^{{\left( {\alpha + \sum\nolimits_{j = 1}^{k} {\beta_{j} x_{ji} } } \right)}} }}{{1 + e^{{\left( {\alpha + \sum\nolimits_{j = 1}^{k} {\beta_{j} x_{ji} } } \right)}} }} = \frac{1}{{1 + e^{{\left( {\alpha + \sum\nolimits_{j = 1}^{k} {\beta_{j} x_{ji} } } \right)}} }}$$

In the land change simulation, natural geographical factors, such as topography, elevation, slope, and distance from existing built-up land, directly affect the development and utilization of land, which are the direct driving factors of ecological land change. Population growth, urbanization, economic development, industrial layout and other factors affect changes in urban land use by controlling the expansion of built-up land, which is an indirect driving factor of ecological land change. In this study, we selected four variables, i.e., elevation, slope, distance from built-up land, and growth rate of built-up land, as the independent variables of the regression model. Whether ecological land changes (0 or 1) represent the dependent variable of the regression model.

In the above independent variables, the elevation data directly use DEM data (unit: m), the slope data are calculated from DEM data (unit: degree (°)), and the distance from built-up land is calculated using the Euclidean distance tool in ArcGIS (unit: km). The above three indicators are numerically continuous and raster-type spatial variables. To distinguish and explore the impact of the growth rate of built-up land on ecological land change, the built-up land growth rate index uses categorical variables rather than continuous variables. First, we calculated the average annual growth rate of built-up land in the administrative units of 13 districts (townships and towns) in Zhuhai city from 1991 to 2018. Then, we divided the above-average annual growth rate into five levels, i.e., 0–5%, 5–10%, 10–20%, 20–35% and > 35%, and assigned each level a value of 1, 2, 3, 4 and 5, respectively. We spatialized the rating assignment results of the growth rate of the abovementioned administrative districts onto each grid in the area. Finally, we obtained the four spatial variables of elevation, slope, distance from built-up land and growth rate of built-up land, all of which had a spatial resolution of 30 m.

In the specific regression analysis, we randomly selected 20,000 samples from ecological land in 1991 and then examined the ecological land attributes of the above samples in 2018. If the sample was still ecological land in 2018, the result of the dependent variable was 1. If the sample was no longer ecological land, it received a value of 0. Finally, these 20,000 samples of the input variables (elevation, slope, distance from built-up land and growth rate of built-up land) and output variables (whether the ecological land changed) were used. In SPSS 25.0, the abovementioned modeling process and model accuracy test were completed using the logistic forward stepwise regression tool to determine the main driving factors of ecological land change and the driving mechanism of each factor in Zhuhai.

## Supplementary information

Supplementary Information.
