# Supplementary material for: Spatial–temporal dynamics and driving factor analysis of urban ecological land in Zhuhai city, China
Source: Sci Rep. 2020 Sep 30;10:16174. doi: 10.1038/s41598-020-73167-0 (PMC7527567; doi:10.1038/s41598-020-73167-0)
Supplement: Supplementary file 1 — Supplementary Information. [file 41598_2020_73167_MOESM1_ESM.doc]

# Supplementary materials for Spatial-temporal dynamics and driving factor analysis of urban ecological land in Zhuhai city, China

Yunfeng Hu^1*,2^, Yunzhi Zhang^1*,2^

1 State Key Laboratory of Resources and Environmental Information System, Institute of Geographic Sciences and Natural Resources Research, CAS, Beijing 100101, China.

2 College of Resources and Environment, University of Chinese Academy of Sciences, Beijing 100049, China.

*Co-correspondence: huyf@lreis.ac.cn; zhangyunzhi@lreis.ac.cn. Tel.: +86-10-64888020

## Supplemental Materials S1: Land use types and descriptions

With reference to the Zhuhai land use planning map, urban characteristics, human activities and land use status and natural ecosystem types, we identified 10 land use types: woodland, grassland, rainfed cropland, paddy fields, aquaculture areas, reservoirs and pit ponds, tidal flats, rivers and shallow water, construction land and unutilized land. The specific descriptions and definitions of all land use types are as follows:

**Table S1.** Land use types and their descriptions^1^.

| Number | Land use types | Description |
| --- | --- | --- |
| 1 | Woodland | Land where trees are grown, including arbor, shrub, bamboo, and forestry use. |
| 2 | Grassland | Land covered by herbaceous plants, including natural pastures and artificial grasslands. |
| 3 | Rainfed cropland | Cropland for cultivation without water supply and irrigation facilities; cropland that has a water supply and irrigation facilities and where dry farming crops are planted; cropland where vegetables are planted; fallow land. |
| 4 | Paddy fields | Cultivated land for crops; such land has a guaranteed water source and irrigation facilities for planting aquatic crops such as rice and lotus root. |
| 5 | Aquiculture areas | Manually excavated or naturally formed pit-water surfaces for aquaculture and corresponding ancillary facilities. |
| 6 | Reservoirs and pit ponds | Constructed reservoirs for water reservation and small natural ponds. |
| 7 | Tidal flats | Tidal flats between the normal water level and flood level of a river or lake; the tidal zone between the high tide level and low tide level along the coast. |
| 8 | Rivers and shallow water | Rivers, including canals; natural lakes; shallow waters along the coast. |
| 9 | Construction land | Land used for urban and rural settlements, factories, and transportation facilities. |
| 10 | Unutilized land | Land that is not put into practical use or that is difficult to use. |

## Supplemental Materials S2: Parameter of the driving factors for modeling

We have summarized the list of possible drivers of ecological land change and established a number of independent variables, such as elevation, slope, the distance from construction land, and of the growth rate of construction land (five categories), to explain and evaluate the changes in ecological land during the study period.

**Table S2.** Parameter table of the driving factors of ecological land change in Zhuhai city from 1991 to 2018.

| Variable | Parameter estimation (β) | Standard error (SE) | Wald χ2 statistics | PR>χ2 | EXP(β) |
| --- | --- | --- | --- | --- | --- |
| Constant | -0.853 | 0.032 | 707.869 | 0.000 | 0.426 |
| Slope (°) | 0.069 | 0.005 | 209.685 | 0.000 | 1.061 |
| Elevation (m) | 0.033 | 0.001 | 631.877 | 0.000 | 1.030 |
| Distance from construction land (km) | 0.473 | 0.014 | 1070.991 | 0.000 | 1.560 |
| Growth rate category 4 | -1.079 | 0.046 | 557.105 | 0.000 | 0.340 |
| Growth rate category 5 | -0.963 | 0.068 | 202.899 | 0.000 | 0.382 |

Table S4 shows that regional construction land growth rate categories 1, 2, and 3 have not entered the forward stepwise regression model. Slope, elevation, the distance from construction land and construction land growth rate categories 4 and 5 are the main driving factors of ecological land change. In the logistic regression, Wald χ2 represents the relative weight of each variable and can be used to predict the contribution of each variable to an event. During the 1991-2018 period, the distance from construction land had the greatest impact on the ecological land change in Zhuhai city, followed by elevation. The regression coefficients of the three explanatory variables of slope, elevation, and the distance from the construction land are positive. According to the event probability formula, it is deduced that there is a negative correlation between the above three variables and the probability of ecological land change. That is, as the distance from construction land increases, elevation and slope increase, and the probability of ecological land change decreases. When slope increases by 1°, the invariant probability of ecological land increases by 1.061 times; when elevation increases by 1 m, the probability increases by 1.030; and when the distance from construction land increases by 1 km, the probability increases by 1.560. In addition, the impact of construction land growth rate categories 4 and 5 on ecological land use is also relatively significant. The rapid growth of construction land, affected by urban expansion pressure, is also a major factor in changes in ecological land use.

## Supplemental Materials S3: Details of the Landsat TM/ETM+/OLI satellite data

The Landsat TM/ETM+ and Landsat OLI data for 1991, 2000, 2010 and 2018 used in the study were obtained from the USGS website (https://earthexplorer.usgs.gov/). The detailed description of the data is as follows^2^:

**Table S3.** Details of the Landsat TM/ETM+/OLI satellite data used in the study.

| Time | Sensors | Row and column number | Description |
| --- | --- | --- | --- |
| 1991 | TM | 122/44  122/45  121/45 | LT05_L1TP_122044_19911117_20170125_01_T1  LT05_L1TP_122045_19911117_20170125_01_T1  LT05_L1TP_121045_19911009_20170125_01_T1 |
| 2000 | ETM+ | 122/44  122/45  121/45 | LE07_L1TP_122044_20001101_20170209_01_T1  LE07_L1TP_122045_20001101_20170209_01_T1  LE07_L1TP_121045_20001228_20170208_01_T1 |
| 2010 | TM | 122/44  122/45  121/45 | LT05_L1TP_122044_20100326_20161016_01_T1  LT05_L1TP_122045_20100326_20161016_01_T1  LT05_L1TP_121045_20101029_20161012_01_T1 |
| 2018 | OLI | 122/44  122/45  121/45 | LC08_L1TP_122044_20180212_20180222_01_T1  LC08_L1TP_122045_20180212_20180222_01_T1  LC08_L1TP_121045_20171203_20171207_01_T1 |

## Supplemental Materials S4: Landscape indicators and descriptions

This study explores the evolution of landscape patterns at the class and landscape levels. The indicators at the class level include five indicators: the patch density (PD), landscape shape index (LSI), largest patch index (LPI), and area-weighted mean contiguity index (CONTIG_AM). The indicators at the landscape level include seven indicators: the edge density (ED), PD, LSI, LPI, contagion (CONTAG), Shannon’s diversity index (SHDI), and Shannon’s evenness index (SHEI). The calculation methods and ecological meanings of the landscape indicators are as follows^3^:

**Table S4.** Landscape indicators and their descriptions.

| Landscape indicators | Formula | Description |
| --- | --- | --- |
| Edge density (ED) | $ED=E/A$ E is the total length of the edge in the landscape (m); A is the landscape area (m^2^) | The ED describes the length of the edge per unit area, making it easy to compare landscapes of different sizes. |
| Patch density (PD) | $PD=N/A$ N is the number of all patches; A is the landscape area (m^2^) | The PD reflects the degree of fragmentation of the landscape and the degree of spatial heterogeneity of the landscape. To a certain extent, it reflects the degree of human interference in the landscape. The larger the PD is, the higher the degree of fragmentation, and the greater the degree of spatial heterogeneity. |
| Largest patch index (LPI) | $LPI=\frac{\max\left(a_{ij}\right)}{A}$ a_ij_ is area of patch ij (m^2^) | The LPI reflects the degree of landscape type dominance; a higher LPI value indicates that this type of landscape has a large degree of dominance. This indicator is used to identify the dominant plaque within the evaluation range. |
| Landscape shape index (LSI) | $LSI=\frac{0.25E}{\sqrt{A}}$ E is the total length of the edge in the landscape (m); A is the landscape area (m^2^) | The LSI reflects the complexity of the shape of the overall landscape. The closer the LSI is to 1, the simpler the overall shape of the landscape. That is, the LSI involves rules or approximate squares; the larger the LSI, the more complicated it is. |
| Area-weighted mean contiguity index (CONTIG_AM) | $\begin{matrix} CONTING\_AM= \\ \sum_{j=1}^{n} \left[\frac{\left[\sum_{r=1}^{z} C_{ijr}/a_{ij}^{*}\right]-1}{V-1}\left(\frac{a_{ij}}{\sum_{j=1}^{n} a_{ij}}\right)\right] \end{matrix}$ | The CONTIG_AM reflects the spatial connectivity or proximity of cells within the plaque of the grid unit, thereby indicating the plaque boundary configuration and the index of the plaque shape. |
| Contagion (CONTAG) | $\begin{matrix} CONTAG=1+\sum_{i=1}^{m} \sum_{k=1}^{m} \left[P_{i}\frac{g_{ik}}{\sum_{k=1}^{m} g_{ik}}\right] \\ \left[\ln\left(P_{i}\frac{g_{ik}}{\sum_{k=1}^{m} g_{ik}}\right)\right]/2\ln(m) \end{matrix}$ | CONTAG reflects the degree of aggregation and extension of different patch types in the landscape; a high CONTAG value indicates that a certain dominant component in the landscape forms good connectivity. |
| Shannon’s diversity index (SHDI) | $SHDI=-\sum_{i=1}^{m} \left(P_{i}\ln P_{i}\right)$ | SHDI reflects the number of landscape components that compose the landscape and the proportion of each landscape component. The greater the value is, the higher the diversity. |
| Shannon’s evenness index (SHEI) | $SHEI=-\sum_{i=1}^{m} \left(P_{i}\ln P_{i}\right)/\ln\left(m\right)$ | SHEI reflects the uniformity of the distribution of different landscape components in the landscape, and it is an important aspect of the diversity index. |

## [References](javascript:;)
